# Optimizing Oral Targeted Anticancer Therapies Study for Patients With Solid Cancer: Protocol for a Randomized Controlled Medication Adherence Program Along With Systematic Collection and Modeling of Pharmacokinetic and Pharmacodynamic Data

**DOI:** 10.2196/30090

**Published:** 2021-06-29

**Authors:** Carole Bandiera, Evelina Cardoso, Isabella Locatelli, Antonia Digklia, Khalil Zaman, Antonella Diciolla, Valérie Cristina, Athina Stravodimou, Aedo Lopez Veronica, Ana Dolcan, Apostolos Sarivalasis, Aikaterini Liapi, Hasna Bouchaab, Angela Orcurto, Jennifer Dotta-Celio, Solange Peters, Laurent Decosterd, Nicolas Widmer, Dorothea Wagner, Chantal Csajka, Marie Paule Schneider

**Affiliations:** 1 School of Pharmaceutical Sciences University of Geneva Geneva Switzerland; 2 Institute of Pharmaceutical Sciences of Western Switzerland University of Geneva Geneva Switzerland; 3 Center for Primary Care and Public Health (Unisanté) University of Lausanne Lausanne Switzerland; 4 Center for Research and Innovation in Clinical Pharmaceutical Sciences Lausanne University Hospital and University of Lausanne Lausanne Switzerland; 5 Department of Oncology Lausanne University Hospital and University of Lausanne Lausanne Switzerland; 6 Service of Clinical Pharmacology Lausanne University Hospital and University of Lausanne Lausanne Switzerland; 7 School of Pharmaceutical Sciences University of Lausanne Lausanne Switzerland; 8 Pharmacy of the Eastern Vaud Hospitals Rennaz Switzerland

**Keywords:** neoplasms, medication adherence, oral anticancer therapies, interprofessional program, adherence electronic measure, pharmacokinetics, pharmacodynamics, NONMEM, motivational interviewing

## Abstract

**Background:**

The strengthening or substitution of intravenous cytotoxic chemotherapy cycles by oral targeted anticancer therapies, such as protein kinase inhibitors (PKIs), has provided impressive clinical benefits and autonomy as well as a better quality of life for patients with cancer. Despite these advances, adverse event management at home and medication adherence remain challenging. In addition, PKI plasma concentrations vary significantly among patients with cancer receiving the same dosage, which could explain part of the observed variability in the therapeutic response.

**Objective:**

The aim of this optimizing oral targeted anticancer therapies (OpTAT) study is to optimize and individualize targeted anticancer treatments to improve patient care and self-monitoring through an interprofessional medication adherence program (IMAP) combined with measurement PKI plasma concentrations.

**Methods:**

The OpTAT study has two parts: (1) a 1:1 randomized medication adherence program, in which the intervention consists of regular motivational interviewing sessions between the patient and the pharmacist, along with the delivery of PKIs in electronic monitors, and (2) a systematic collection of blood samples and clinical and biological data for combined pharmacokinetic and pharmacodynamic analysis. On the basis of the electronic monitor data, medication adherence will be compared between groups following the three operational definitions: implementation of treatment during the persistent period, persistence with treatment and longitudinal adherence. The implementation will be described using generalized estimating equation models. The persistence of PKI use will be represented using a Kaplan-Meier survival curve. Longitudinal adherence is defined as the product of persistence and implementation. PKI pharmacokinetics will be studied using a population approach. The relationship between drug exposure and efficacy outcomes will be explored using Cox regression analysis of progression-free survival. The relationship between drug exposure and toxicity will be analyzed using a pharmacokinetic-pharmacodynamic model and by logistic regression analysis. Receiver operating characteristic analyses will be applied to evaluate the best exposure threshold associated with clinical benefits.

**Results:**

The first patient was included in May 2015. As of June 2021, 262 patients had participated in at least one part of the study: 250 patients gave at least one blood sample, and 130 participated in the adherence study. Data collection is in process, and the final data analysis is planned to be performed in 2022.

**Conclusions:**

The OpTAT study will inform us about the effectiveness of the IMAP program in patients with solid cancers treated with PKIs. It will also shed light on PKI pharmacokinetic and pharmacodynamic properties, with the aim of learning how to adapt the PKI dosage at the individual patient level to increase PKI clinical suitability. The IMAP program will enable interprofessional teams to learn about patients’ needs and to consider their concerns about their PKI self-management, considering the patient as an active partner.

**Trial Registration:**

ClinicalTrials.gov NCT04484064; https://clinicaltrials.gov/ct2/show/NCT04484064.

**International Registered Report Identifier (IRRID):**

DERR1-10.2196/30090

## Introduction

### Background

On the basis of the Global Cancer Observatory estimates, cancer was responsible for almost 10 million deaths globally in 2020 [1], making it a leading cause of death [2]. In Switzerland, more than 40,000 new patients are diagnosed with cancer each year [3]; however, the mortality rate of many cancers is decreasing, mainly because of the implementation of both screening programs and novel targeted and immune-related therapies [4].

For 15 years, the strengthening or alternatively the substitution of intravenous cytotoxic chemotherapy cycles by oral, sometimes long-term, targeted anticancer therapies has offered impressive clinical benefit and autonomy as well as a better quality of life for patients with cancer. Targeted cancer therapies involve protein kinase inhibitors (PKIs) and inhibitors of growth factor receptors, which are used to treat a variety of cancers, including gastrointestinal stromal tumors; kidney, thyroid, colorectal, lung, and breast cancers; melanoma; and sarcomas. Although treatment failure and poor tolerance are common in clinical practice, they cannot all be attributed to solid tumor multidrug resistance, as they can also be triggered by suboptimal medication adherence or inadequate drug plasma concentrations.

Suboptimal medication adherence is one of the causes of cancer progression and higher costs for health systems [5-7]. Thus, supporting patient drug self-management is key to achieving the best clinical outcome. In the past decade, interventional studies have focused mainly on patients with breast cancer treated with hormonal treatments and patients with chronic myeloid leukemia using imatinib. Evaluations of interventions to support PKI adherence in other solid cancers are scarce. In a systematic review, adherence to oral anticancer therapies was estimated to vary between 46% and 100% [8]. This review addressed adherence to various classes of oral anticancer therapies using various measures of adherence among patients with solid cancers, multiple myeloma, and chronic leukemia. Adherence estimation is not often reliable, as it is frequently evaluated through a moderate-quality methodology using subjective self-report questionnaires [9-11]. The link between longitudinal medication adherence, clinical factors, quality of life, survival, and cancer progression has been underinvestigated in the literature [12].

Since 1995, a routine interprofessional medication adherence program (IMAP) has been implemented at the University Community Pharmacy of the Center for Primary Care and Public Health Unisanté (Lausanne, Switzerland) to support drug self-management in patients with chronic illness [13]. Before initiating the optimizing oral targeted anticancer therapies (OpTAT) study, we conducted a feasibility study in which the included patients were mostly diagnosed with gastrointestinal stromal tumors or breast cancer. Adherence was estimated for several classes of oral anticancer therapies (capecitabine, letrozole, exemestane, imatinib, sunitinib, and temozolomide). We showed that patients with cancer agreed to participate and adopted the program; 12-month persistence was estimated at 85%, and the 12-month implementation rate was 97% in persistent patients [14].

Oral PKIs are licensed at a fixed dose despite the variable plasma concentrations observed in real-life situations [15,16]. This substantial interpatient pharmacokinetic variability may be explained by demographic (eg, age, body weight, and sex) and environmental factors (eg, concomitant medication and smoking), relevant physiopathological conditions (eg, organ failure and albumin levels) [17,18], genetic polymorphisms of metabolic enzymes or drug transporters, and patient behavior (eg, medication adherence) [12]. The characterization of pharmacokinetic variability is essential in oncology, given the risk of under- or overexposure, which may contribute to insufficient efficacy or undesirable toxicity in patients with cancer, respectively. Moreover, considerable efforts remain to be undertaken to characterize the correlation between drug plasma concentrations and the therapeutic response of most PKIs and to define the therapeutic targets associated with clinical benefits in the context of therapeutic drug monitoring [19].

### Objectives

The global aims of the OpTAT study are to establish the necessary knowledge to build rational monitoring strategies through medication adherence monitoring and patient-adjusted dosage in real-life conditions, which should contribute to optimizing the therapeutic benefit of PKIs, in order to improve outpatient cancer care.

The primary objectives are to better characterize the prospective and longitudinal patterns of PKI adherence in patients participating in a medication adherence program versus a control group and to identify key driver-modifiable adherence factors.

The secondary objectives are to quantify interpatient variability, identify sources of variability in serum PKI levels, and characterize the relationships between the serum concentration, the therapeutic response and the toxicity of these treatments in a population of patients with solid cancer.

The hypothesis states that the medication adherence program will increase PKI implementation and persistence before the treatment is stopped for any reasons beyond the patient’s control or therapeutic change compared with the control group. Moreover, it is hypothesized that interpatient variability in PKI plasma concentrations plays a fundamental role in the heterogeneity of the therapeutic response and may play a role in PKI adherence.

## Methods

### Ethical Considerations

The local ethics committee (Vaud, Switzerland) approved the OpTAT study in April 2015. Since then, two amendments have been accepted in July 2017 and October 2020. The study will be conducted in accordance with the current version of the Declaration of Helsinki and international good clinical practice principles. The protocol was peer-reviewed by five experts in April 2017 (Multimedia Appendix 1), leading to the founding of the study by the Swiss Cancer Research Foundation. This protocol was written according to the SPIRIT (Standard Protocol Items: Recommendations for Interventional Trials) guidelines (Multimedia Appendix 2) [20].

### Trial Design

**Figure 1:**
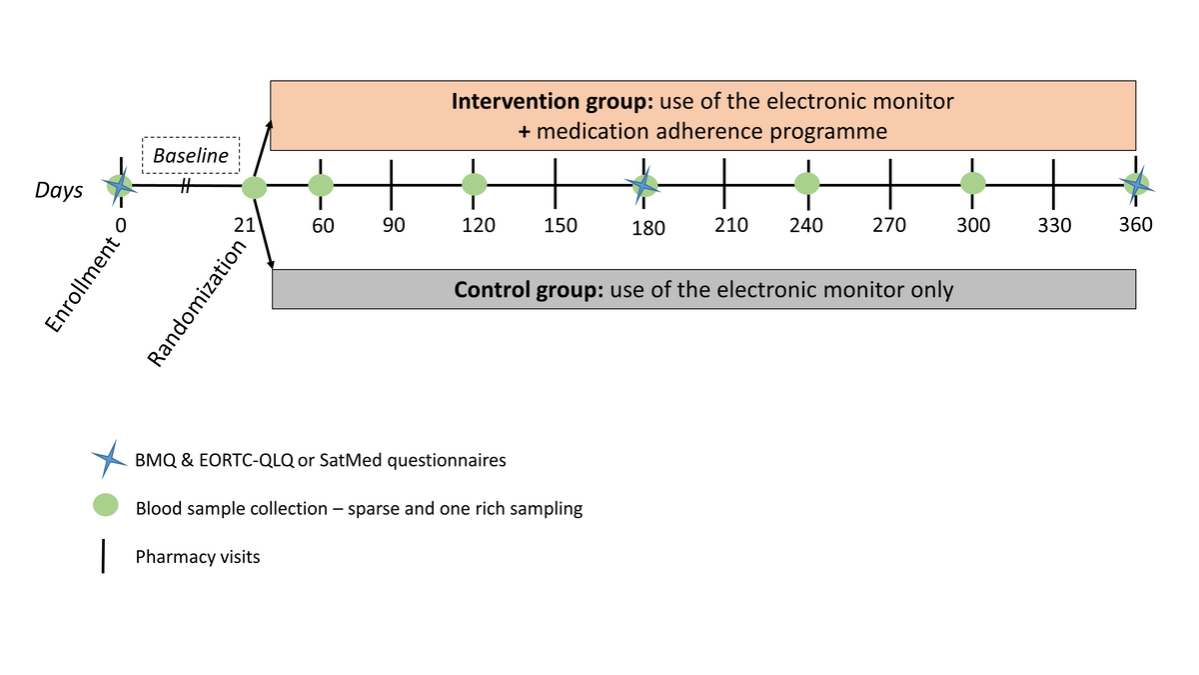
Design of the optimizing oral targeted anticancer therapies study. BMQ: Belief about Medicines Questionnaire; EORTC-QLQ: European Organization for Research and Treatment of Cancer Quality of Life Questionnaire; SatMed: Treatment Satisfaction with Medicines Questionnaire.

This monocentric prospective study comprises two parts: (1) an open randomized controlled medication adherence study and (2) an observational pharmacokinetic and pharmacodynamic study. This design (Figure 1) explores whether medication adherence evolves differently in each group and explores the interplay between adherence and PKI pharmacokinetic parameters.

### Enrollment of Participants

This translational study will be conducted at the Medical Oncology Service of the Department of Oncology of the Lausanne University Hospital (CHUV) outpatient clinic (Lausanne, Switzerland) in collaboration with the Community Pharmacy of the Center for Primary Care and Public Health Unisanté (Lausanne, Switzerland) and the Center for Research and Innovation in Clinical Pharmacy Sciences (CHUV).

Adult patients aged >18 years who were treated with a PKI for solid tumors and followed at the CHUV were eligible to participate in this study. Participants with an inability to make decisions, with cognitive disorders, under tutelage, or not fluent in French or English or without the aid of an interpreter will be excluded from the study. Patients will be excluded solely from the medication adherence part of the study if they do not self-manage oral anticancer treatment (ie, nursing homes and home care services) or if they are already enrolled in an interventional study. If one PKI is switched to another PKI during the study, the patient will continue to participate in the study. However, if the PKI treatment is stopped or changed to a non-PKI anticancer treatment, the participant exits the OpTAT study; but, the data collected are kept for analysis.

The OpTAT study is voluntary, and patients will need to sign an informed consent form to participate in the medication adherence study, the pharmacokinetic and pharmacodynamic study, or both. The recruitment of participants began in May 2015. It ended in March 2021 for the medication adherence part but is ongoing until December 2021 for the pharmacokinetics part.

Oncologists were asked to refer eligible patients to a pharmacist PhD student or to the research staff from the Center of Experimental Therapeutics (CHUV), who will present the informed consent form to the patient after the medical visit. The participants will follow their treatment as usual, and no changes in the usual medical procedure will be made. No payment or compensation will be provided to the patients or to the oncologists as the patients are being seen as part of their routine follow-up clinical care.

### Randomized Controlled Medication Adherence Study

#### Instruments: Use of the Electronic Monitors MEMS and Questionnaires in Both Groups

All patients participating in the medication adherence study will use an electronic monitor (MEMS and MEMS AS, AARDEX Group) for 12 months or until PKI is stopped. The electronic monitor registers the date and time of each opening, and a liquid-crystal display monitor on top of the cap indicates the number of daily openings to the patient. All patients included in the study will be asked to complete two validated questionnaires at three timepoints: inclusion, 6 months postinclusion, and at the end of the study. The first questionnaire is the *Belief about Medicines Questionnaire*, which assesses the patient’s cognitive representation of medication [21]. The second is the *European Organization for Research and Treatment of Cancer Quality of Life Questionnaire* (version 3.0), which assesses the quality of life of patients with cancer [22]. During the initial months of the study implementation, the patients with cancer filled out the *Treatment Satisfaction with Medicines Questionnaire* [23]; however, it was switched to the *European Organization for Research and Treatment of Cancer Quality of Life Questionnaire*, as the latter was more adapted to patients treated with a PKI. The questionnaires were translated and validated in French, with satisfactory psychometric properties [24-26].

#### Repeated Medication Adherence Interviews and Feedback (Intervention Group)

Patients included in the intervention group will visit the routine IMAP of the pharmacy of Unisanté. They will attend 15- to 30-minute medication adherence interviews, which will take place after each clinical appointment over 12 months. On the basis of the information-motivation-behavior model embedded in sociocognitive theory [27], the pharmacist will explore in depth the home drug self-management skills, side effect management, motivation, self-efficacy, and consider cofactors such as depression, anxiety, and drug or alcohol addiction. Patients’ beliefs about PKIs will be explored, and information about the PKI will be delivered and discussed at each visit. Determinants of and gaps in medication adherence will be discussed with the pharmacist in an empathetic and nonjudgmental way using motivational interviewing skills.

The electronic monitor data will be uploaded to MEDAMIGO software (MEMS and MEMS AS), which generates graphs of electronic monitor daily openings. Feedback on adherence to PKI therapy, based on the information generated by the electronic monitor, will be provided to and discussed with the patient. Interprofessionality is a cornerstone of the program, and pharmacists will summarize the intervention in a semistructured report sent to the oncologist after each intervention. During the COVID-19 pandemic, motivational interviews will be conducted by phone calls with patients considered by clinicians to be at a high risk of SARS-CoV-2 infection. All pharmacists who perform the medication adherence intervention must have attended specific training to lead motivational interviews (two full-day medication adherence initial training and continuous education based on the debriefing of a motivational interview with a patient every 12-18 months) [13].

#### Control Group

Patients included in the control group will use an electronic monitor but will be considered standard-of-care patients as they will not receive any medication adherence intervention. The electronic monitor opening data of the control group will be concealed from the patients, pharmacy team, clinicians, and investigators until the end of the study. If an oncologist expressively asks for adherence data because of stringent and urgent clinical matters, the patient will be excluded from the OpTAT study and referred to the routine medication adherence program of the Community Pharmacy of the Center for Primary Care and Public Health Unisanté.

#### Assignment of the Intervention

The collection of adherence data patterns in both groups at baseline is crucial to check whether adherence patterns are homogeneous between the groups before the intervention starts. In both groups, patients will use an electronic monitor for at least 21 days before randomization. At the end of the baseline period, the patients will be randomized 1:1 to either attend the medication adherence program (intervention group) or be part of the standard of care (control group; Figure 1). The randomization lists will stratify participants according to their cancer type and the time elapsed between PKI initiation and the time of inclusion (less or more than 30 days). The randomization sheet lists 1 (intervention group) or 0 (control group) and will be provided by an independent researcher from the Unisanté Research Support Unit. The randomization list established by Excel (version 2016, Microsoft) will be based on variable size block randomization to prevent predictability and ensure a balanced allocation to each group. After randomization, the patient, research team, pharmacy, and clinical team will all be aware of the assigned group.

### Observational Pharmacokinetic and Pharmacodynamic Study

A total of 10 mL of peripheral blood samples will be collected for up to a maximum of 8 samples per patient or until PKI is stopped at an unselected time after the last PKI intake. The blood samples will be centrifuged, the plasma will be frozen and stored until PKI levels are measured, and the dry pellet will be analyzed for the exploration of genetic variants involved in PKI pharmacokinetics. Moreover, for patients who accept, 6 × 5 mL blood samples will be collected over a maximum of 12 hours during one visit to the hospital. Specific information on pharmacokinetic data will be collected at each blood draw, and all relevant clinical and biological information will be obtained from the medical files of the patients (detailed in the *Collection and Management of Data* section).

### Outcomes

#### Primary Outcomes: Randomized Controlled Medication Adherence Study

The primary outcome was global adherence, defined as the percentage of days with correct medication use of each subject. The sample size was calculated to ensure sufficient power to test the difference in the mean logit global adherence between groups (see the *Sample Size* section). Medication adherence will also be compared between groups following the three operational definitions defined by the EMERGE (European Society for Patient Adherence, Compliance, and Persistence Medication Adherence Reporting Guideline) guidelines: implementation of treatment during the persistent period (daily management of oral targeted anticancer drugs), persistence with treatment (the distribution of times between enrollment and discontinuation), and longitudinal adherence (the product of persistence and implementation) [14,28,29].

#### Secondary Outcomes: Pharmacokinetic and Pharmacodynamic Data

The plasma concentrations of the PKI and active metabolites, if appropriate, will be measured using validated methods of liquid chromatography-tandem mass spectrometry for each blood sample collected [30,31]. Internal standards will be used to optimize the analytical accuracy and precision of the method, essentially by abrogating the influence of matrix effects and inherent variability in the extraction procedure. The pharmacokinetic variables quantifying PKI exposure will then be calculated from population-based individual Bayesian pharmacokinetic parameters and expressed as the area under the curve and trough drug concentrations (minimum drug concentration) [32]. The efficacy of anticancer drugs will be expressed as progression-free survival, which is the time interval between PKI initiation and the date of tumor progression, defined according to the standardized criteria *Response Evaluation Criteria In Solid Tumors* [33], or death from any cause. If a patient is event-free (progression or death), the time will be censored at the last follow-up visit, the end of the study follow-up, or treatment discontinuation. The response to treatment will be assessed according to local standards, usually every 3 months, using a computed tomography scan or positron emission tomography scan. In addition, an overall survival analysis will be undertaken in an exploratory manner. The toxicity of PKI will be assessed according to the National Cancer Institute–Common Terminology Criteria for Adverse Events [34].

### Sample Size

#### Randomized Controlled Medication Adherence Study

We assumed that the mean longitudinal adherence will remain stable at 95% in the intervention group during the follow-up period, whereas it would decrease from 95% to 80% in the control group during the 340 days from randomization to the end of follow-up. We also assumed an SD for the logit of longitudinal adherence of 1.8, for both groups and at all timepoints. This corresponds to 95% individual adherence in the intervals 10%-99.3% and 35%-99.9% in the control and intervention groups, respectively, at the end of follow-up. A sample size calculation was performed to simulate individual series of daily medication (1=at least the correct number of daily openings of the electronic monitor; 0=fewer daily openings than prescribed) according to the above parameters to consider both the interindividual variability (SD 1.8) and the intraindividual variability (the measurement error). Considering that the number of measures of a subject will be 340 and assuming a 10% dropout in the middle of the year, 120 patients must be included in the study (60 in each group) to reach a power of 80% with a .05 significance level when comparing the mean of the logit of global adherence between groups using a two-tailed Student *t* test. Differences are considered statistically significant if *P*<.05.

#### Pharmacokinetic and Pharmacodynamic Study

It has been empirically determined that at least 50 patients with several samples per PKI are necessary to characterize drug pharmacokinetics and variability.

### Collection and Management of Data

In collaboration with the investigators, the community pharmacy of Unisanté will manage the PKI adherence data, and the Center of Experimental Therapeutics will manage the blood samples as well as the clinical, biological, and sociodemographic data. The schedule of enrollment, intervention, and assessments is shown in Multimedia Appendix 3. Sociodemographic and relevant clinical and biological information regarding the evaluation of the disease will be obtained from the administrative and electronic patient medical records (Soarian, Cerner). The data will be registered prospectively in the REDCap (Research Electronic Data Capture; Vanderbilt University) platform, a secure web application for research data collection, providing a unique study identification number for each patient. To ensure confidentiality and data quality, only principal investigators will have access to this file. After the analysis, the database will be registered in the secured data repository of the University of Geneva (Yareta). Patient blood samples will be stored in the Center of Experimental Therapeutics, and the health data will be stored in the data warehouse of the CHUV for a maximum of 15 years after the end of the study before destruction.

### Data Analyses

#### Medication Adherence Analysis

Adherence to PKI will be assessed using electronic monitor, calculated pill counts, and patient reports. Medication behavior will be described with a binary variable (1=at least the correct number of daily openings of the electronic monitor; 0=fewer daily openings than prescribed) in each group. This coding method is commonly used; however, this code cannot discriminate overadherence, which is considered in the same way as optimal adherence. The implementation will be described in each group using generalized estimating equation models; persistence with PKI (ie, adherence until PKI discontinuation) will be represented using a Kaplan-Meier survival curve [14]. Global adherence will be estimated in each group with the proportion of patients with correct medication taking, defined as ≥95% days with correct dosing during days 1 to 360, compared between groups using a chi-square test. As ≥95% days with the correct dosing threshold is an assumption, we will perform sensitivity analysis with other thresholds such as 90%, 85%, and 80%. Multivariate logistic regression models will be used to evaluate whether any detected effect of the intervention remained after adjusting for potential sociodemographic or clinical confounders. As a secondary analysis, the difference in medication taking will be explored across genders.

A systematic computerized term search in the adherence reports of the intervention group will be performed to identify the most frequent variables affecting PKI adherence, and a regression model will be used to discover their association with adherence [35].

Sociodemographic data and quality of life and beliefs about medication of patients who refused to participate and completed both questionnaires versus those who accepted to participate in the adherence study will be compared at baseline. For patients included in the adherence study, the results of the questionnaires will be compared at randomization, 6 months, and at the end of the study. Changes in *Belief about Medicines Questionnaire* and *European Organization for Research and Treatment of Cancer Quality of Life Questionnaire* or the *Treatment Satisfaction with Medicines Questionnaire* will be described longitudinally for the included patients and transversely per group at 6 and 12 months. Medians and IQRs or proportions will be used as appropriate. Nonparametric tests (eg, Mann-Whitney-Wilcoxon) or Fisher exact test of two proportions will be used to test the differences between groups.

Ultimately, the adherence patterns and their relationship with drug exposure and clinical response will be evaluated in an exploratory analysis.

To minimize interpretation bias, the statistician will not be aware of the group assignments during the analysis. The data will be analyzed in an intention-to-treat manner. All statistical analyses will be performed using the R statistical package (version 3.6, The R Foundation for Statistical Computing) [36].

#### Population Pharmacokinetic Analysis

Population pharmacokinetic analyses will be performed using nonlinear mixed regression models with the software program NONMEM (Icon Development Solutions). This approach allows for the characterization of the average pharmacokinetic profile of PKI from data pooled over all sampled individuals and the quantification of inter- and intraindividual variability. This approach allows us to analyze sparse (few drug concentrations per patient) and unbalanced data. The built models integrate active metabolites when appropriate. The impact of potential sources of variability will be evaluated using linear and nonlinear functions.

#### Relationships Between PKI Exposure, Efficacy, and Toxicity

The correlation between PKI exposure and efficacy will be explored using Cox regression analysis of progression-free survival. The correlation between PKI exposure and toxicity will be explored by pharmacokinetic and pharmacodynamic models using the software program NONMEM or by logistic regression analysis. Receiver operating characteristic analyses will be used to evaluate the best exposure threshold to predict clinical outcomes and performance (in terms of area under the receiver operating characteristics curve and sensitivity and specificity).

#### Missing Data

Missing data will not be imputed but will be clearly identified and considered during the analysis process (nature and frequency) and described in the publication.

#### Patient and Public Involvement

Patients or the public will not be involved in the design, conduct, reporting, or dissemination plans of this research. The participants will be informed about the study results after the final publication using lay language.

## Results

The first patient was included in May 2015. Until June 2021, 262 patients have participated in at least one part of the study (250 patients have given at least one blood sample, and 130 have been included in the adherence study). In June 2021, 51 patients treated with palbociclib have provided at least one blood sample, and the adherence study currently includes 43 participants with breast cancer, which corresponds to most of the included participants. Moreover, so far, at least one blood sample has been collected from patients treated with 31 different PKIs and their combination (Multimedia Appendix 4).

Data collection is in process, and the final analysis will be performed in 2022. The results of the study will be submitted to local, national, and international conferences and to peer-reviewed open access journals for publication.

## Discussion

### Strength of the Study

The OpTAT study is based on a mixed methodology involving a randomized controlled medication adherence study, along with pharmacokinetic and pharmacodynamic data collection. The methodology is robust, as adherence data will be assessed through electronic monitoring, often considered the gold standard, in both the intervention and control groups. Participants randomized to the intervention group will be included in a routine medication adherence program (IMAP) that has previously been shown to lead to significant improvements in terms of medication adherence; clinical outcomes; and retention in care of other chronically ill patients, such as those with HIV [13,14,37-39]. Moreover, the association between longitudinal adherence and clinical data as well as quality of life and beliefs about medicines through questionnaires will be explored, and the literature in this area is scarce.

The second strength of this study is the pharmacokinetic analysis of a wide range of PKIs. PKIs are marketed under a single regimen according to *the one dosage fits all patients* paradigm, which may be an important source of side effects that are potentially detrimental to the continuation of the course of the treatment and patient adherence. Hence, our ultimate and cutting-edge development is to combine an analysis of PKI adherence, pharmacokinetics, and pharmacodynamics.

### Limitations of the Study

This study has some limitations. First, the adherence data will be collected over 12 months, a time frame during which several stops and breaks in the treatment regimen can occur. This will be a challenge for data analysis; however, the adherence team and the statistician, who will perform the analysis, have already dealt with this kind of data and are experts in this field [14]. Second, we expect some variability in the frequency of adherence visits among participants. Indeed, the adherence interviews are scheduled after each patient’s clinical appointment, and the frequency can vary from a quarterly to a monthly visit depending on the organization of the clinic and the severity of the patient’s disease. Third, the use of an electronic monitor can introduce a Hawthorne effect during the first 6 weeks of the study, which disappears afterward [40]. This effect is explained by the fact that patients who know they are being observed may improve their usual medication behavior. The impact of this effect will be explored in the analysis. Fourth, the number of blood samples may not be sufficient to initiate a population pharmacokinetic analysis for each monitored PKI. All limitations will be considered while analyzing the results and will be detailed in the final publication.

### Conclusions

The OpTAT study will strengthen knowledge on the effectiveness of the routine IMAP medication adherence program in patients with solid cancers treated with PKIs. The IMAP will enable interprofessional teams to learn about patients’ needs and concerns about their PKI self-management. The study will also shed light on PKI pharmacokinetics and its relationship with pharmacodynamic properties, with the aim of adapting dosage regimens at the individual patient level, increasing the clinical suitability of PKIs.

Adherence combined with pharmacokinetic data will inform pharmacists and clinicians on how to support patients during the entire course of their PKI treatment and how to manage adaptations of treatment efficiently, with the patient as an informed partner.

## Appendix

Multimedia Appendix 1 Peer-review reports from the Scientific Office of the Swiss Cancer League/Swiss Cancer Research.

Multimedia Appendix 2 SPIRIT (Standard Protocol Items: Recommendations for Interventional Trials) checklist completed for the optimizing oral targeted anticancer therapies protocol.

Multimedia Appendix 3 Schedule of enrollment, intervention, and assessments of the optimizing oral targeted anticancer therapies study.

Multimedia Appendix 4 Protein kinase inhibitors and their combination included in the optimizing oral targeted anticancer therapies study.

## Abbreviations

CHUV: Lausanne University Hospital
EMERGE: European Society for Patient Adherence, Compliance, and Persistence Medication Adherence Reporting Guideline
IMAP: Interprofessional medication adherence program
OpTAT: Optimizing oral targeted anticancer therapies
PKI: Protein kinase inhibitor
REDCap: Research Electronic Data Capture
SPIRIT: Standard Protocol Items: Recommendations for Interventional Trials
