# Supplementary material for: Optimizing Oral Targeted Anticancer Therapies Study for Patients With Solid Cancer: Protocol for a Randomized Controlled Medication Adherence Program Along With Systematic Collection and Modeling of Pharmacokinetic and Pharmacodynamic Data
Source: JMIR Res Protoc. 2021 Jun 29;10(6):e30090. doi: 10.2196/30090 (PMC8278299; doi:10.2196/30090)
Supplement: Multimedia Appendix 1 [file resprot_v10i6e30090_app1.pdf]

Scientific Office
Swiss Cancer League / Swiss Cancer Research
Effingerstrasse 40
P.O. Box 8219
CH-3001 Bern
t +41 31 389 91 16
f +41 31 389 91 62
scientific-office@swisscancer.ch
www.swisscancer.ch/research

Health Services Research Review

**Project-Number**	HSR-4077-11-2016
**Principal Investigator**	Dr Marie Paule Schneider
**Requested Sum**	248,942.00
**Reviewer**	4

1. Review

2. Critical Comments

Note: The detailed critical comments will be passed on to the project applicant without mention of the name of the reviewer.
Please provide us with your **detailed critical or supportive comments on the following aspects** of the proposed project:

1. Relevance to improve health services in oncology
2. Scientific quality / adequacy of proposed research methods
3. Feasibility of project by the PI's group
4. Past accomplishments of the PI

Dr. Schneider and colleagues will implement a program that supports patient compliance with oral anticancer drugs (protein kinase inhibitors), and monitors compliance as well as drug concentrations and treatment toxicity. PKI is a class of oral anticancer drugs that is frequently used in oncology, the drugs are often given over extended periods of time, and their use is often complicated by complex and cumulative toxicity that complicates patient management. Oncologists and pharmacologists are well aware of the substantial variability of clinical tolerability and outcome in patients receiving PKI's, and this is in part explained by variability in individual drug pharmacokinetics. The project outlined by Dr. Schneider and colleagues is highly relevant to improve the quality of cancer care, it adapts well accepted and adequate methodology to approach study objectives. Additionally, the authors performed a pilot study strongly supporting the feasibility of the current study, and the project submitted to KLS continues well in line with the focus of the research group. The investigators have a sound track record in the field of pharmaco-oncology, therapeutic drug monitoring of anticancer drugs and compliance with anticancer systemic treatment. Detailed comments: The authors randomized patients into a control and an intervention treatment arm to study the impact of a 12-month medication adherence program on adherence and treatment outcome. As all patients are getting a so called 'MEMS device' to monitor daily tablet intake, a minor bias is introduce in the control arm, as the disposal of the MEMS device per se may be seen as a 'therapeutic intervention' to support treatment adherence. Still monitoring of drug intake by using a MEMS device is generally seen as the 'gold standard' and is supported by the Reviewer. Additionally, the investigators are aware of this intervention ('Hawthorne bias') and have adressed this in their study outline. Allocation of human resources to the study period until 2022 is well elaborated and reasonable. The project team is highly qualified to execute this complex clinical trial. The second study hypothesis is defined as 'improved self-management of PKI-related adverse events and longer persistence on PKI at 4,6, and 12 months due to the study intervention'. At this point, it seems important to assess which parameters will define 'patient self-management' as this is a soft study endpoint. Depending on the 'patient mix', i.e. the tumor diseases included into the study, it is expected that only a minority of patients will receive PKI's for the full study period of 12 months. The investigators should consider that the 'patient mix' will have a substantial impact on treatment duration (e.g. ranging from an average of 2 months in patients with EGFR-wildtype NSCLC on EGFR inhibitors to several years in CML patients on e.g. imatinib, dasatinib or nilotinib). For pharmacokinetic blood sampling, the investigators should prefer through level

sampling, as PKI through plasma concentrations are usually seen as the relevant PK parameter. Mathematical modeling may be used to simulate drug through concentrations, but PK sampling should be done before drug intake if ever possible. The sample size and power calculation is sound and clear; it takes into account a certain proportion of 'drop outs', and these numbers are estimated from a previous pilot study.

Scientific Office
Swiss Cancer League / Swiss Cancer Research
Effingerstrasse 40
P.O. Box 8219
CH-3001 Bern
t +41 31 389 91 16
f +41 31 389 91 62
scientific-office@swisscancer.ch
www.swisscancer.ch/research

Health Services Research Review

**Project-Number**	HSR-4077-11-2016
**Principal Investigator**	Dr Marie Paule Schneider
**Requested Sum**	248,942.00
**Reviewer**	5

1. Review

2. Critical Comments

Note: The detailed critical comments will be passed on to the project applicant without mention of the name of the reviewer.
Please provide us with your **detailed critical or supportive comments on the following aspects** of the proposed project:

1. Relevance to improve health services in oncology
2. Scientific quality / adequacy of proposed research methods
3. Feasibility of project by the PI's group
4. Past accomplishments of the PI

1. Relevance to improve health services in oncology Medication adherence determines if oral anti-cancer therapies work or not. Individualized oral anti-cancer therapies efficacy will be dependent on patients adhering to the dosing regimen over an extended period of time. Ample evidence in chronic illness in general and the cancer population in particular highlights that medication non-adherence is substantial and negatively impacts on clinical and economic outcomes. This project is therefore most timely as it aims at testing if an adherence support program is effective to improve adherence in patients on protease kinase inhibitors (PKI). Moreover this project aims at substantiating specific pharmacokinetic and pharmacodynamic parameters of PKI treatment given varying levels of adherence. 2. Scientific quality / adequacy of proposed research methods The study's strength lies in the solid assessment of medication adherence using electronic monitoring, the most reliable assessment method to date. The intervention tested is reflecting the current state of science of adherence enhancing interventions using behavioural change techniques that are proven to be efficacious. The choice of a RCT design to test the efficacy of the intervention is appropriate. The sample size calculation is using correct parameters and the sample size proposed has sufficient power. It would be valuable to get some more information in the proposal how the researcher will handle their modelling for the 2 other research aims. Will they do this modelling using the control arm data for this purpose and not consider the intervention arm data? Or how have they considered to deal with the intervention's effect on the parameters of interest in their modelling? 3. Feasibility of project by the PI's group The research group is excellently positioned to do this work. They have the expertise in view of adherence research (MPS), pharmacological modelling (CC) and oncology (CC & ADW). MPS has extensive experience in running an adherence management program at the outpatient clinic pharmacy at the CHUV and has extensive clinical connections to carry out this study. CC has performed a series of modelling studies using electronic monitoring data also preparing the team well to analyse the vast amount of data this study will generate. ADW will provide the oncological clinical experience for this project. The team also collaborate with Westrock the company providng the MEMS caps to assess medication adherence electronically. Together they build a team with the needed track record and competencies to succeed in answering their aims. 4. Past accomplishments of the PI MPS has a vast clinical and research background in the field of medication adherence research and clinical practice. She is author of 31 publications and has obtained several research grants in the field of adherence. She has continued training in research methods as shown by a DAS in clinical

research which she completed in 2016. She runs an medication adherence program at the pharmacy of the outpatient clinical of the CHUV and has demonstrated a strong translational focus in her professional portfolio, a guarantee that this project not only generates interesting data yet also will provided added value for oncology patients who are on PKI regimens. I recommend to provide funding to this project

Scientific Office
Swiss Cancer League / Swiss Cancer Research
Effingerstrasse 40
P.O. Box 8219
CH-3001 Bern
t +41 31 389 91 16
f +41 31 389 91 62
scientific-office@swisscancer.ch
www.swisscancer.ch/research

Health Services Research Review

**Project-Number**	HSR-4077-11-2016
**Principal Investigator**	Dr Marie Paule Schneider
**Requested Sum**	248,942.00
**Reviewer**	6

1. Review

2. Critical Comments

Note: The detailed critical comments will be passed on to the project applicant without mention of the name of the reviewer.
Please provide us with your **detailed critical or supportive comments on the following aspects** of the proposed project:
1. Relevance to improve health services in oncology
2. Scientific quality / adequacy of proposed research methods
3. Feasibility of project by the PI's group
4. Past accomplishments of the PI

To investigate patiens' adherence to oral anti-cancer drugs (protein kinase inhibitors) and to relate this to drug levels appears relevant and innovative. This may provide inputs for patient education and thus it may contribute to improvement of cancer treatment, including individualized patient care and support. The scientific quality is given and the proposed research methods appears convincing. Thus the feasibility of the project is provided, including the past accomplishments of the PI. The required amount of funding appears reasonable. One point which could be added in the project, is a psychological co-worker who may interview patients about their difficulties related to medication and to find out more about hidden aspects concerning insufficient adherence, or unmet needs of these severely ill patients.

Scientific Office
Swiss Cancer League / Swiss Cancer Research
Effingerstrasse 40
P.O. Box 8219
CH-3001 Bern
t +41 31 389 91 16
f +41 31 389 91 62
scientific-office@swisscancer.ch
www.swisscancer.ch/research

Health Services Research Review

**Project-Number**	HSR-4077-11-2016
**Principal Investigator**	Dr Marie Paule Schneider
**Requested Sum**	248,942.00
**Reviewer**	7

1. Review

2. Critical Comments

Note: The detailed critical comments will be passed on to the project applicant without mention of the name of the reviewer.
Please provide us with your **detailed critical or supportive comments on the following aspects** of the proposed project:

1. Relevance to improve health services in oncology
2. Scientific quality / adequacy of proposed research methods
3. Feasibility of project by the PI's group
4. Past accomplishments of the PI

1. Relevance to improve health services in oncology The authors aim at improving adherence to oral anticancer treatment with protein kinase inhibitors. Obviously, adherence to the drug treatment is essential in all chronic disease but even more in cancer treatment. Appropriate blood levels can only be achieved with the frequent uptake of the appropriate dose. Especially in PKI the individual uptake and in consequence blood level can variate tremendously between different individuals and therefore non-compliance can be caused by toxic blood concentrations. On the other hand, treatment success needs appropriate drug levels and compliance. Interventions which may increase the adherence to cancer drugs are likely to have huge impact on the treatment success, namely survival rate and time. Authors should also focus on assessing what patients characteristics determined the "improved-self-management". This could be helpful for the implementation of this new knowledeg in clinical practice lateron. 2. Scientific quality / adequacy of proposed research methods 3. The authors present a well described study with a clear description of the study aim (hypothesis) as well as the primary and secondary outcomes. The intervention is also well described and based on existing evidence regarding the improvement of adherence-enhancing interventions. They also present a plausible sample size calculation. 4. Feasibility of project by the PI's group 5. According to the patients treated at the oncology department of the CHUV with PKIs (80-110 p.a.) and the calculated patient sample of 152, the feasibility seems to be given. The team can be trusted to conduct such a RCT based on previous experience and expertise. 4. Past accomplishments of the PI The PI has a specific research background in adherence and can be regarded as expert in the field. Based on the expertise of the PI and the whole team, a successful conducted study can be expected.

Scientific Office
Swiss Cancer League / Swiss Cancer Research
Effingerstrasse 40
P.O. Box 8219
CH-3001 Bern
t +41 31 389 91 16
f +41 31 389 91 62
scientific-office@swisscancer.ch
www.swisscancer.ch/research

Health Services Research Review

**Project-Number**	HSR-4077-11-2016
**Principal Investigator**	Dr Marie Paule Schneider
**Requested Sum**	248,942.00
**Reviewer**	8

1. Review

2. Critical Comments

Note: The detailed critical comments will be passed on to the project applicant without mention of the name of the reviewer.
Please provide us with your **detailed critical or supportive comments on the following aspects** of the proposed project:

1. Relevance to improve health services in oncology
2. Scientific quality / adequacy of proposed research methods
3. Feasibility of project by the PI's group
4. Past accomplishments of the PI

1. This is a highly relevant study in clinical oncology with an ever bigger significance in the future with more oral PKIs entering the clinical field and market. 2. Triangulation of medication adherence, PKI dosage adjustment and plasma drug concentration corresponds to the clinical need. The study flow based on a pilot study (without clearly stated results) demonstrates feasibility and adaptation to clinical reality at the oncology service of CHUV. Sample size calculation reflects current literature and assumptions. 3. The group has demonstrated in the past its ability to conduct clinical trials as well to develop methods and techniques to be one of the leading institutions in terms of patient adherence and drug concentration-time profiles. 4. The PI is a leading patient adherence researcher on an international level.
