# Supplementary material for: Optimizing Oral Targeted Anticancer Therapies Study for Patients With Solid Cancer: Protocol for a Randomized Controlled Medication Adherence Program Along With Systematic Collection and Modeling of Pharmacokinetic and Pharmacodynamic Data
Source: JMIR Res Protoc. 2021 Jun 29;10(6):e30090. doi: 10.2196/30090 (PMC8278299; doi:10.2196/30090)
Supplement: Multimedia Appendix 3 [file resprot_v10i6e30090_app3.docx]

### Multimedia Appendix 3: Schedule of enrollment, intervention and assessments of the optimizing oral targeted anticancer therapies study

| **OpTAT study period** | | | | | | | | | | | |
| --- | --- | --- | --- | --- | --- | --- | --- | --- | --- | --- | --- |
| **Description of the event** | | **Enrollment** | **Allocation** | **Post allocation** | | | | | | **Closeout/End of study** | **Collected by:** |
| **Time point (days)** | t-1 | t0  [d0] | t1  [d21] | t2  [d60] | t3  [d90] | t4  [d120] | t5  [d150] | t6  [d180] | tx_+1_  [dx_+30_] | t12  [d360] |  |
| **Recruitment data collection** | | | | | | | | | | | |
| **Eligibility screen** | x |  |  |  |  |  |  |  |  |  | Oncologists, CB, EC, research staff |
| **In case of patient refusal to participate to the adherence study:**  **-Reasons for refusal (descriptive)**  **-Questionnaires^a^** | x |  |  |  |  |  |  |  |  |  | CB, EC, research staff |
| **Informed consent** |  | x |  |  |  |  |  |  |  |  | CB, EC, research staff |
| **Medication adherence data collection** | | | | | | | | | | | |
| **Randomization** |  |  | x |  |  |  |  |  |  |  | Pharmacists |
| **Intervention group:**  **Open-reading of adherence EM data, adherence conciliation by pill-count and patients’ report^b^, semistructured interviews, adherence report sent to the clinical team** |  |  | x | x | x | x | x | x | x | x | Pharmacists, technicians |
| **Control group:**  **Double-blind reading of adherence EM data, patients’ report^b^ about EM use** |  |  | x | x | x | x | x | x | x | x | Pharmacists, technicians |
| **Questionnaires^a^ to be filled in both groups** |  | x |  |  |  |  |  | x |  | x | Pharmacists, technicians |
| **Pharmacokinetic data collection at each blood draw** | | | | | | | | | | | |
| **Blood draws (maximum 8 per patient)** |  | x |  | x |  | x |  | x | x | x | Nurses in the oncology unit |
| **Specific data collected for the pharmacokinetic analysis^c^** |  | x | x | x | x | x | x | x | x | x | Research staff |
| **Sociodemographic and clinical data** | | | | | | | | | | | |
| **Sociodemographic variables^d^** |  | x |  |  |  |  |  |  |  |  | CB, EC |
| **Clinical variables^e^** |  | x | x | x | x | x | x | x | x | x | CB, EC |

^a^BMQ (Belief about Medicines Questionnaire) and EORTC-QLQ (European Organization for Research and Treatment of Cancer Quality of Life Questionnaire) or SatMed (Treatment Satisfaction with Medicines Questionnaire)

^b^At each pharmacy visit in both groups, the pharmacist checks the EM use though a set of validation questions (i.e., if the medication is prepared in advance for later use, nonmonitored periods).

^c^Information collected in a laboratory report form (LRF): PKI name, dosage and regimen, dates of cycles if applicable and notification if the treatment is discontinued, date and hour of the last PKI intake, PKI taken on an empty stomach or not

^d^Age, civil status, gender, ethnicity, nationality

^e^Cancer diagnosis, date of the cancer diagnosis, cancer stage and grade, PKI treatment line and objective, PKI start date, concomitant oncologic treatment at inclusion, previous oncologic treatment before inclusion, progression date during PKI use, best clinical response according to “Response Evaluation Criteria In Solid Tumors” (RECIST), death date, cotreatments at inclusion, use of alternative medicines at inclusion, adverse effects during PKI use.
