# Supplementary material for: Optimizing Oral Targeted Anticancer Therapies Study for Patients With Solid Cancer: Protocol for a Randomized Controlled Medication Adherence Program Along With Systematic Collection and Modeling of Pharmacokinetic and Pharmacodynamic Data
Source: JMIR Res Protoc. 2021 Jun 29;10(6):e30090. doi: 10.2196/30090 (PMC8278299; doi:10.2196/30090)
Supplement: Multimedia Appendix 4 [file resprot_v10i6e30090_app4.docx]

### Multimedia Appendix 4: PKIs and their combination included in the optimizing oral targeted anticancer therapies study

| Afatinib |
| --- |
| Alectinib |
| Alpelisib |
| Axitinib |
| Binimetinib/Encorafenib |
| Cabozantinib |
| Cobimetinib |
| Cobimetinib/Vemurafenib |
| Crizotinib |
| Erlotinib |
| Everolimus |
| Gefitinib |
| Imatinib |
| Lapatinib |
| Lenvatinib |
| Lorlatinib |
| Niraparib |
| Olaparib |
| Osimertinib |
| Pazopanib |
| Palbociclib |
| Regorafenib |
| Ribociclib |
| Rociletinib |
| Sorafenib |
| Sorafenib/Everolimus |
| Sunitinib |
| Trametinib |
| Trametinib/Dabrafenib |
| Trametinib/olaparib |
| Vemurafenib |
